# Umbilical endosalpingiosis: a case report

**DOI:** 10.1186/1752-1947-4-287

**Published:** 2010-08-24

**Authors:** Theodossis S Papavramidis, Konstantinos Sapalidis, Nick Michalopoulos, Georgia Karayannopoulou, Angeliki Cheva, Spiros T Papavramidis

**Affiliations:** 1 Department of Surgery, AHEPA University Hospital, Aristotle University of Thessaloniki, Thessaloniki, Greece; 2 Department of Pathology, AHEPA University Hospital, Aristotle University of Thessaloniki, Thessaloniki, Greece

## Abstract

**Introduction:**

Endosalpingiosis describes the ectopic growth of Fallopian tube epithelium. Pathology confirms the presence of a tube-like epithelium containing three types of cells: ciliated, columnar cells; non-ciliated, columnar secretory mucous cells; and intercalary cells.

We report the case of a woman with umbilical endosalpingiosis and examine the nature and characteristics of cutaneous endosalpingiosis by reviewing and combining the other four cases existing in the international literature.

**Case presentation:**

A 50-year-old Caucasian, Greek woman presented with a pale brown nodule in her umbilicus. The nodule was asymptomatic, with no cyclical discomfort or variation in size. Her personal medical, surgical and gynecologic history was uneventful. An excision within healthy margins was performed under local anesthesia. A cystic formation measuring 2.7×1.7×1 cm was removed. Histological examination confirmed umbilical endosalpingiosis.

**Conclusions:**

Umbilical endosalpingiosis is a very rare manifestation of the non-neoplasmatic disorders of the Müllerian system. It appears with cyclic symptoms of pain and swelling of the umbilicus, but not always. The disease is diagnosed using pathologic findings and surgical excision is the definitive treatment.

## Introduction

Endosalpingiosis is a rare clinical entity that describes the ectopic growth of Fallopian tube epithelium [1]. Endosalpingiosis, endometriosis and endocervicosis constitute the triad of non-neoplastic disorders of the Müllerian system. These pathologies are found in isolation, but are more commonly found in association with one another [2,3]. The diagnosis of these pathologies is made histologically. In the case of endosalpingiosis, pathology confirms the presence of a tube-like epithelium containing three types of cells: ciliated, columnar cells; non-ciliated, columnar mucous secretor cells; and the so-called intercalary or peg cells [4,5].

We report the case of an adult woman with umbilical endosalpingiosis and elucidate the nature and characteristics of cutaneous endosalpingiosis by reviewing and combining the four cases existing in the international literature.

## Case presentation

**Figure 1:**
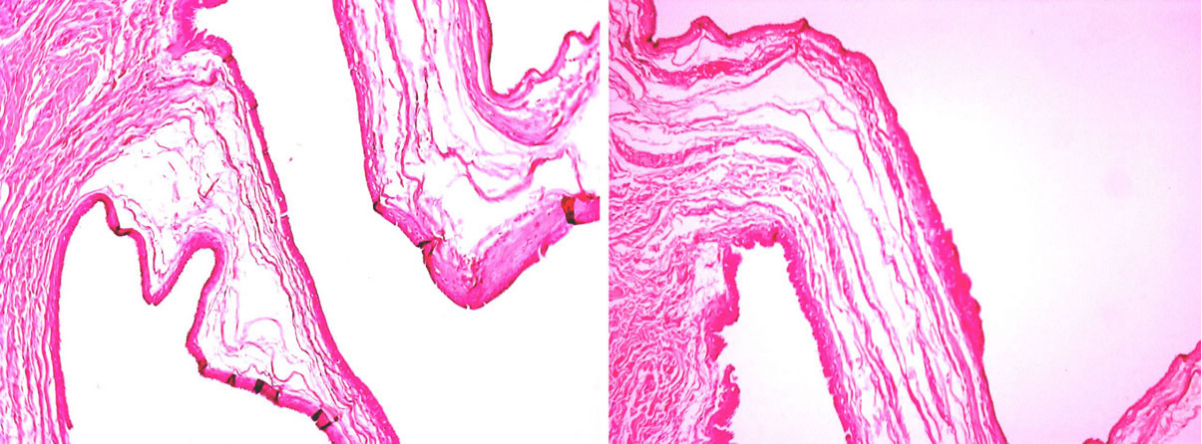
**Unilocular cyst with papillary projections into the lumen (Hematoxylin and Eosin ×4)**.

**Figure 2:**
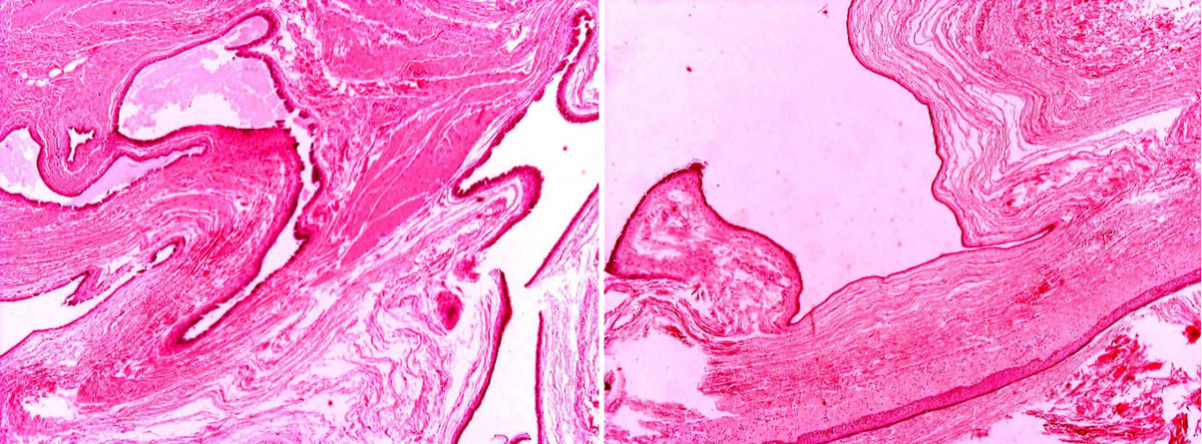
**Unilocular cyst with papillary projections into the lumen (Hematoxylin and Eosin ×10)**.

**Figure 3:**
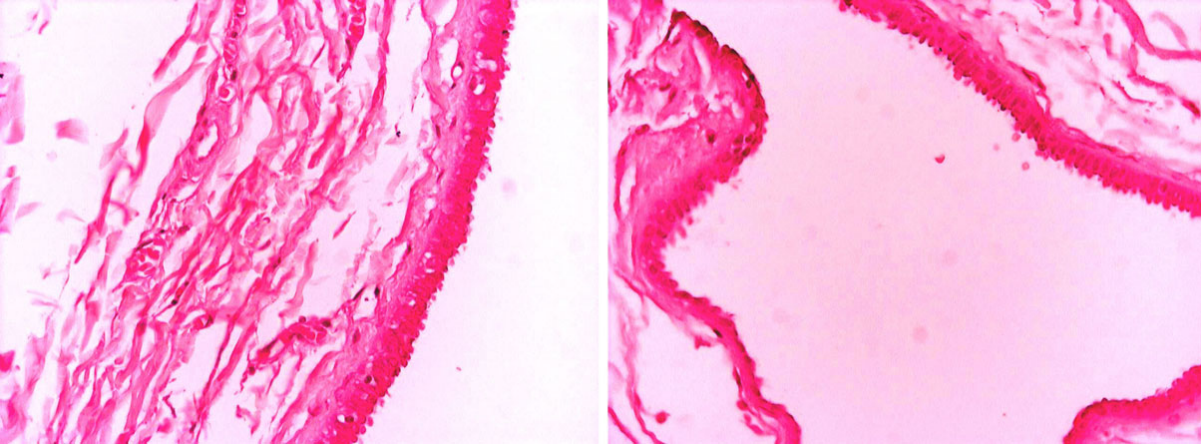
**The lining of the cyst consisted of epithelium cells cuboidal and ciliate (Hematoxylin and Eosin ×40)**.

**Figure 4:**
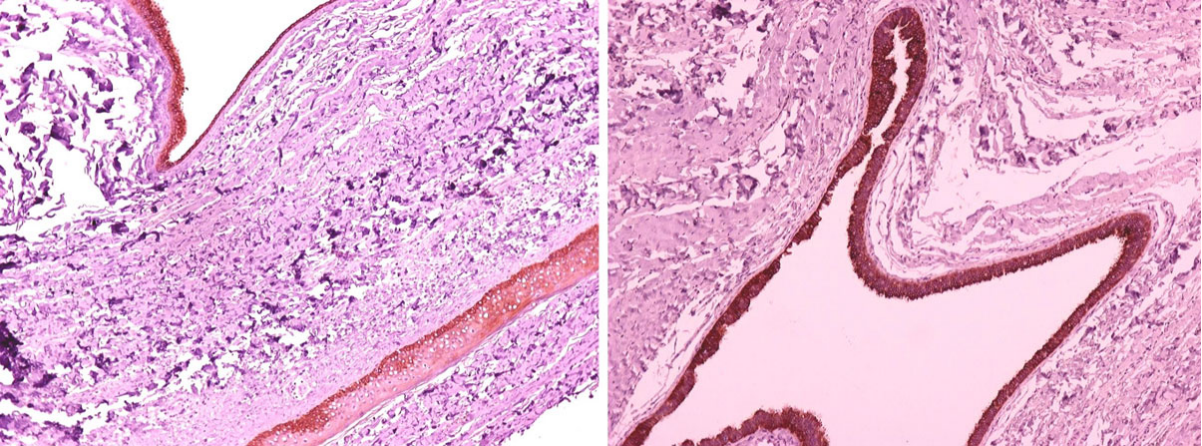
**Immunohistochemical staining shows positivity for keratins AE1/AE3 (Keratins AE1/AE3 ×40)**.

A 50-year-old Caucasian, Greek woman presented with a pale brown nodule in her umbilicus. The nodule was asymptomatic, with no cyclical discomfort or variation in size. Her personal medical, surgical and gynecologic history was uneventful. An excision within healthy margins was performed under local anesthesia. A cystic formation measuring 2.7×1.7×1 cm was removed. There were no signs of malignancy and no evidence of endometrial component in her surrounding tissue. Pathologic examination showed a unilocular cyst with papillary projections into the lumen (Figures 1 and 2). The cyst was surrounded by edematous fibrous tissue. The lining consisted of epithelium cells, both cuboidal and ciliate (Figure 3). Immunohistochemical staining showed positivity for keratins AE1/AE3 (Figure 4). Finally, a histological examination posed the diagnosis of cutaneous endosalpingiosis.

## Discussion

The term endosalpingiosis was employed for the first time by Sampson *et al*. in 1930. Under that term, the author designated any unusual growth and invasion of tubal epithelium in tubal stumps, in subjects who had undergone previous salpingectomy or tubal sterilization [1]. The different theories for the pathogenesis of endosalpingiosis are similar to those for endometriosis, since those two entities, together with endocervicosis, constitute the non-neoplastic disorders of the Müllerian system. The different models can be traced back to two basic ideas. One group of theories is based on the fact that endometrial cells (or their precursors) are transported by various routes (transtubal, hematogenous, lymphogenous or by direct apposition) and implanted in the affected organ. The other group of theories suggests that Müllerian ectopias are the result of metaplastic processes in the target organ (coelomic metaplasia theory, secondary Müllerian system) or from scattered embryonic rest [6-8]. We believe that the first group of theories is inadequate when explaining the origin of endosalpingiosis in our case report, since she has a free gynecologic, obstetric and surgical history. In our case report, it is likely that the Müllerian ectopia resulted either from metaplastic processed or scattered embryonic rest.

Non-neoplastic glandular proliferation showing spontaneous Müllerian differentiation has been described in many sites including the vagina [9], uterine cervix [10], urinary bladder [11,12], appendix [13], peritoneum [14,15], abdominal wall (inguinal channel, umbilicus) [2,16], and the lymph nodes [17]. However, to the best of our knowledge, this is the first case of a patient with spontaneous endosalpingiosis presenting as a nodule on the abdominal wall.

The differential diagnosis of nodular umbilical lesions should include a wide range of diseases; such as hernia, keloid, abscess, lipoma, hematoma, sebaceous cyst, adenocarcinoma [primary of metastatic (Sister Joseph nodule)], melanoma, suture granuloma, pyogenic granuloma, desmoid tumor, sarcoma, lymphoma, endometriosis, and endosalpingiosis. Of course, the final diagnosis should be made by pathology [18].

In the international literature, this is the fifth case of umbilical endosalpingiosis. The other four cases refer to patients with previous medical history of gynecologic procedures [5,19,20], while this is the first case of spontaneous appearance. These lesions appear as nodules of the umbilicus and are usually brownish in colour. The main symptoms (besides the esthetic) are pain and size fluctuation with menstruation. The treatment of choice is surgical excision. Our opinion is that excision has to be made under local anesthesia, in order to minimize morbidity and hospitalization. However, the patient has to be notified that in the case of a reappearance of abdominal pain (especially in the lower quadrant), a laparoscopy should be performed in order to exclude abdominal endometriosis.

## Conclusions

Umbilical endosalpingiosis is a very rare manifestation of the non-neoplasmatic disorders of the Müllerian system. Normally, it appears with cyclic symptoms of pain and swelling of the umbilicus, but not always. The disease is diagnosed using pathologic findings and surgical excision is the definitive treatment.

## Consent

Written informed consent was obtained from the patient for publication of this case report and any accompanying images. A copy of the written consent is available for review by the Editor-in-Chief of this journal.

## Competing interests

The authors declare that they have no competing interests.

## Authors' contributions

TSP analyzed and interpreted the patient data and drafted the manuscript. KS received the patient in our out-patient department and was the principal surgeon. NM received the patient in our out-patient department, was the auxillary surgeon and drafted the manuscript. GK performed the pathological examination and was a major contributor in writing the manuscript. AC performed the pathological examination. STP was responsible for the overall treatment of the patient and corrected the manuscript. All authors read and approved the final manuscript.
